# Adhesion-induced chronic abdominal pain: a case report on the diagnostic value of Carnett’s test

**DOI:** 10.1186/s13256-019-2026-7

**Published:** 2019-04-18

**Authors:** Tsunetaka Kijima, Ryoji Hyakudomi, Tatsuya Hashimoto, Akari Kusaka, Toshihiko Nakatani, Yutaka Ishibashi

**Affiliations:** 1 Department of General Medicine, Faculty of Medicine, Shimane University, 89-1, Enyacho, Izumo City, Shimane 693-8501 Japan; 2 Department of Digestive and General Surgery, Faculty of Medicine, Shimane University, 89-1, Enyacho, Izumo City, Shimane 693-8501 Japan; 3 Palliative Care Center, Shimane University Hospital, 89-1, Enyacho, Izumo City, Shimane 693-8501 Japan; 4 Department of Anesthesiology, Faculty of Medicine, Shimane University, 89-1, Enyacho, Izumo City, Shimane 693-8501 Japan; 5 Department of Palliative Care, Faculty of Medicine, Shimane University, 89-1, Enyacho, Izumo City, Shimane 693-8501 Japan

**Keywords:** Chronic abdominal pain, Adhesion, Carnett’s test

## Abstract

**Background:**

Chronic abdominal pain is a common clinical problem. However, diagnosing chronic abdominal pain often requires detailed diagnostic evaluations in addition to sufficient history taking and physical examination, owing to its uncertain etiology.

**Case presentation:**

We report a case of a 36-year-old man with chronic abdominal pain originating from postoperative adhesions. Postoperative adhesions are common phenomena, and abdominal surgery can cause severe abdominal pain, the source of which can be difficult to detect. Carnett’s test is useful to detect abdominal wall tenderness and to determine the affected abdominal quadrant. Incorporating its use with a detailed chronological clinical history contributes to the improvement of diagnostic accuracy. In addition to the above-mentioned information, attention to subtle imaging findings may provide greater diagnostic accuracy.

**Conclusions:**

Abdominal pain induced by postoperative adhesions was reduced by laparoscopic adhesiolysis. Carnett’s test is an effective tool for evaluating pain and detecting its cause.

**Electronic supplementary material:**

The online version of this article (https://doi.org/10.1186/s13256-019-2026-7) contains supplementary material, which is available to authorized users.

## Background

**Fig. 1:**
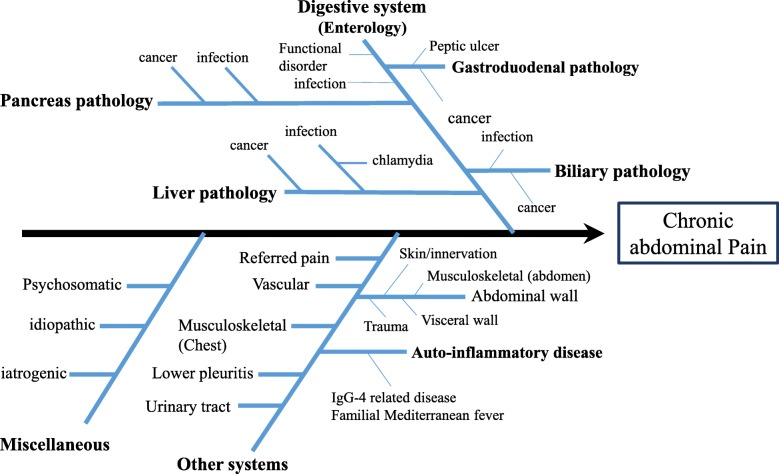
An Ishikawa diagram (fish bone diagram) for a man presenting with a complaint of “chronic abdominal pain”

**Fig. 2:**
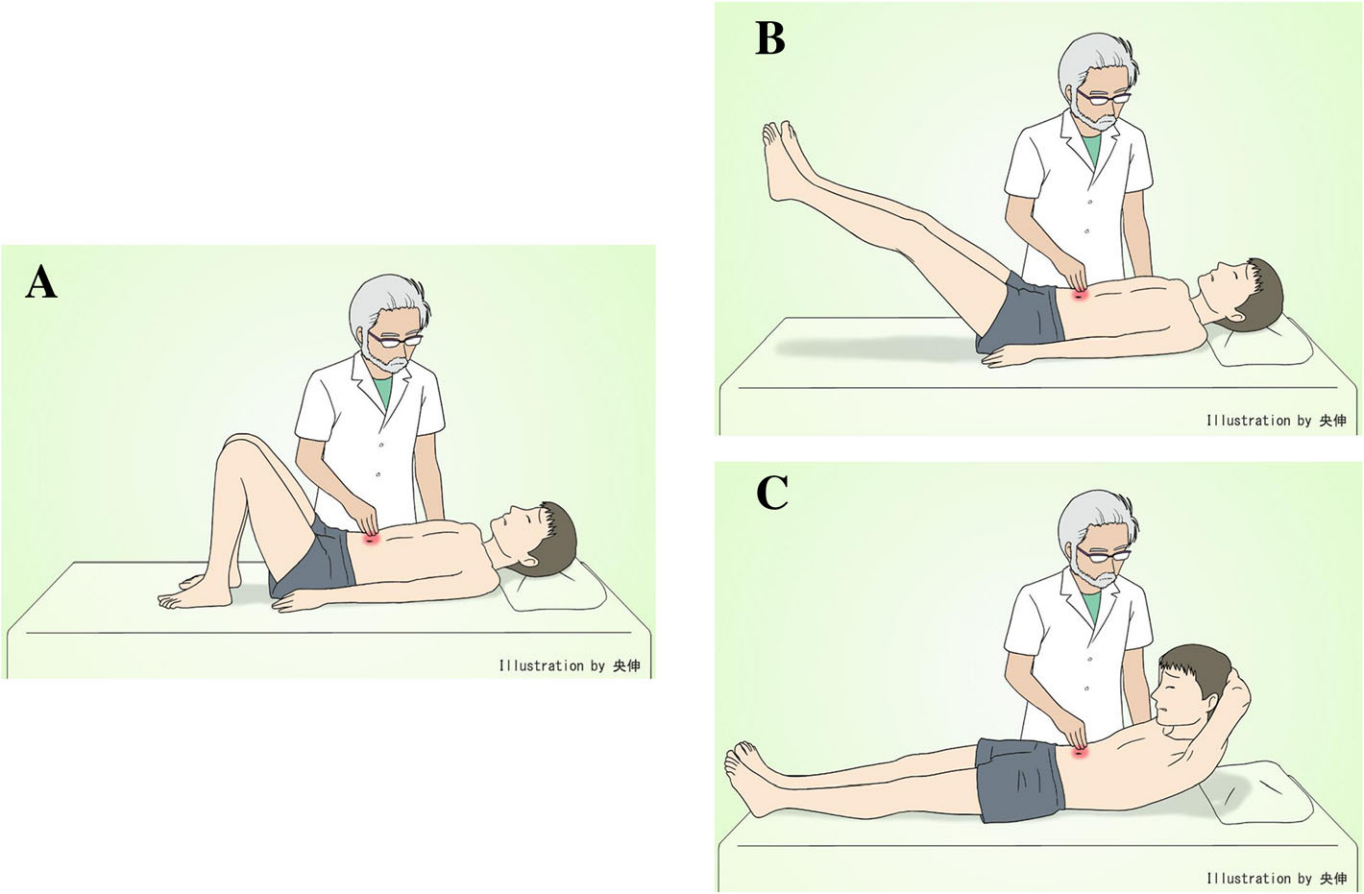
Carnett’s test A (**a**) and Carnett’s test B (**b**, **c**). First, the examiner confirms the area of tenderness with the patient in a relaxed, supine position (**a**). Second, the examiner confirms whether tenderness continues, worsens, or decreases at the same point while the patient raises the legs (**b**) or elevates the head and shoulders (**c**)

Chronic abdominal pain is a relatively common clinical problem that requires detailed diagnostic evaluations, owing to its uncertain etiology. Chronic abdominal pain is associated with many causes, so a systematic approach that considers both anatomical and physiological factors can improve clinical reasoning [1]. An Ishikawa diagram (fish bone diagram) is used to perform clinical reasoning (Fig. 1) and helps to identify the causes of chronic abdominal pain associated with digestive system diseases (for example, chronic pancreatitis [2], functional gastrointestinal disorders [3]), the skin/innervation (for example, abdominal cutaneous nerve entrapment syndrome [4]), musculoskeletal causes (including referred pain from the spine, such as nerve irritation caused by slipping rib syndrome or disorders of the thoracic spine [5]), abdominal wall pain (for example, pain originating in the structure of the abdominal wall [4, 6–8]), visceral wall pain (for example, pain due to pelvic adhesions [9]), vascular conditions (for example, aneurysm [10]), infectious diseases (for example, chlamydial infection [11]), neoplasm (for example, pancreatic cancer [2]), collagen/allergic conditions (for example, immunoglobulin G4 [IgG4]-related disease [12], familial Mediterranean fever [13]), iatrogenic causes (for example, chronic pain due to intraabdominal adhesion after operation [14–17]), and psychosomatic conditions (for example, psychogenic abdominal pain [18, 19]). Among these causes, we focused on abdominal pain that is associated with the skin, nerve, abdominal muscle, and peritoneal wall [4]. This pain has some features that help differentiate it from pain associated with visceral diseases. Similar features include abdominal tenderness, mild appetite loss, and nausea, and different features include pain unrelated to meals or bowel function, pain severity that is possibly related to posture, and tender pain that arises from a location that is only a few centimeters in diameter. Because there are many similarities, abdominal wall pain is likely to be misdiagnosed and often results in inappropriate diagnostic testing, leading to unsatisfactory treatment. Therefore, we focused on Carnett’s test, a useful physical examination to distinguish abdominal wall pain from visceral pain [6]. This test was reported by Carnett in 1926 [20]. It is performed by palpating a limited area of tenderness in a supine, relaxed patient (described as Carnett’s test A; *see* Fig. 2a), which subsequently confirms continued tenderness as the patient tenses the abdominal wall while the head and shoulders are elevated or while raising both legs off the table (described as Carnett’s test B; *see* Fig. 2b and c). Carnett and Greenbaum *et al.* defined the positive sign as follows: If tensed abdominal muscles have almost as much or more tenderness than the relaxed abdominal muscles, it is positive [8, 20]. Carnett hypothesized that if that pain derives from a visceral source, the tensed abdominal muscles protect the underlying structures, and therefore the tenderness should be reduced, whereas continued tenderness during muscle contraction indicates the abdominal wall as the origin of pain [4, 20]. Moreover, Carnett’s test is reported to be useful for diagnosing psychogenic abdominal pain [18]. We report a case of chronic abdominal pain that took 2 years to diagnose and that necessitated two operations.

## Case presentation

A 36-year-old man, originally from Latin America, presented at our outpatient department with complaints of abdominal pain that had persisted for 2 months. The patient had first noticed right lateral abdominal pain 2 months prior to the visit, and the pain was gradually worsening. The abdominal pain was localized in an area ranging from the right upper to the right lateral abdomen. The patient had undergone cholecystectomy for acute cholecystitis as a 32-year-old in Latin America and had moved to Japan for work approximately 3 years prior to his initial visit to our hospital. He had returned to Latin America once about 6 to 7 months before presenting at our clinic. When he went back to Japan, his weight had increased from 130 kg to 145 kg. He did not experience abdominal pain immediately after his return to Japan, but, as noted above, he started to gradually feel pain in the right lateral region about 2 months prior to presentation.

He first visited another hospital emergency department 1 month after onset of the pain. Initially, gastrointestinal tract spasm was suspected, and he was treated with tiquizium bromide. Though the medication partially relieved his abdominal pain, most of the pain persisted. The result of a workup by a urologist was negative, even though nephrolithiasis was suspected. His abdominal pain was exacerbated upon changing posture, and thus it was suspected to be of somatic rather than visceral origin. Abdominal pain persisted despite treatment with loxoprofen sodium hydrate, and any cause of abdominal pain was not detected on further evaluations, including hematologic laboratory analysis, urine analysis, gastroscopy, or abdominal computed tomography (CT). Finally, he was referred to our hospital for further examination.

The results of screening for depression were negative, and the patient did not have symptoms such as loss of interest, depressed feelings, or any specific changes of surrounding conditions, such as family or work environment changes. He had no history of sexually transmitted infection, and his vital signs were within normal limits. His physical examination result was positive for Carnett’s test, and a prior surgical scar of approximately 18 cm was apparent at the right subcostal region. The patient experienced strong pain surrounding the surgical scar that was exacerbated by tapping. There were no skin rashes localized surrounding the pain. His pain exacerbated to 8 on a pain scale when he moved, such as during standing up or rolling over simultaneously. When he stopped moving, pain was partially relieved within 1 minute (3 on a pain scale). When he moved again, abdominal pain was again exacerbated. Hence, he was awakened by the abdominal pain when rolling over. No inflammation was detected (leukocyte count was 8580/mm^3^ and C-reactive protein was 0.10 mg/dl), and other laboratory findings were nonspecific, including liver/kidney function, blood glucose, and electrolytes. Urinary analysis indicated red blood cell count < 1/high-power field, white blood cell count 1–4/high-power field. Additionally, no abnormality was detected for *Chlamydia trachomatis* IgG/IgA, and no abnormality was apparent on the electrocardiogram. Enhanced CT revealed bilateral renal stones and fatty liver.

We first considered abdominal wall pain due to nerve entrapment because the Carnett’s test result was positive; therefore, we scheduled a trigger point injection at the site of tenderness. About 2 weeks later, the patient visited the emergency department of our hospital, reporting that his prior abdominal pain had decreased but that he was experiencing right inguinal pain. Loxoprofen administration had no effect on the pain. Costovertebral angle pain was apparent on tapping, the result of urine analysis was positive for occult blood, and abdominal CT revealed a urinary stone at the right urinary duct to the bladder. After pentazocine hydrochloride was administered for pain relief, the urinary stone was passed the following day. However, the patient’s right lateral abdominal pain was not relieved.

**Fig. 3:**
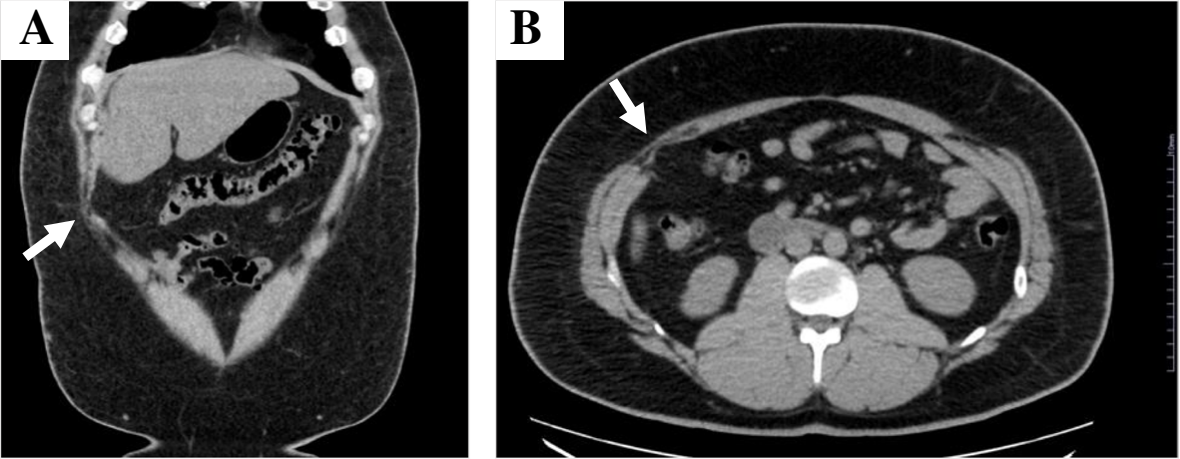
The transverse abdominal muscle shows atrophy at the postoperative scar on plain abdominal coronal computed tomography (**a**) and plain abdominal transverse computed tomography (**b**). *Arrows* indicate adhesive region of the greater omentum

**Fig. 4:**
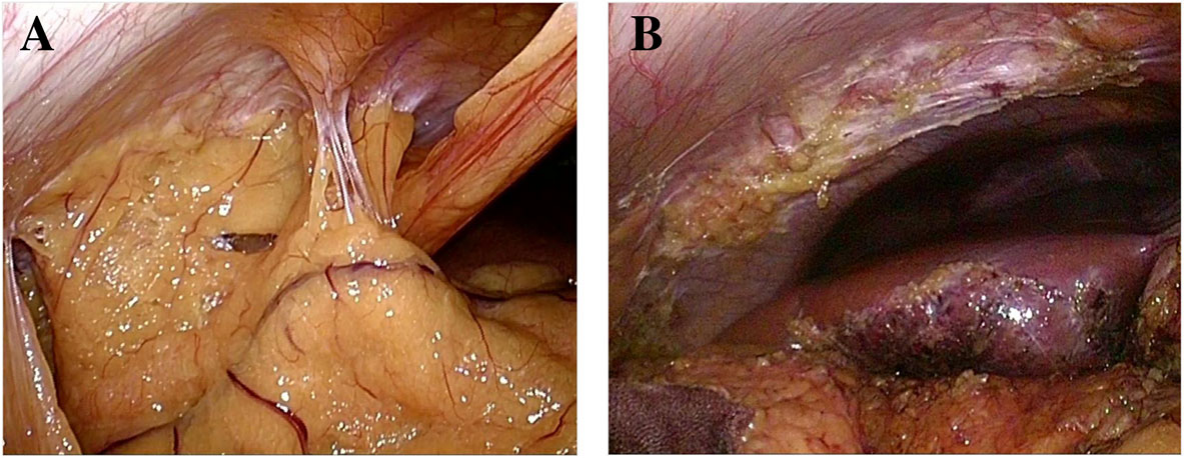
Adhesion was observed between the peritoneum and greater omentum, liver, and ascending colon (**a**). Laparoscopic adhesiolysis was conducted at those adhesive regions (**b**)

He felt that lying in the lateral position mostly relieved his pain. He had occasional vomiting. The abdominal pain was exacerbated by movements, such as rolling over, standing up, walking, and coughing. Injection of 1% xylocaine 10 ml at a trigger point of the right lateral region led to about 30% relief in pain. The patient was referred to an anesthesiologist for further evaluation and treatment, who performed transverse abdominal plane block and administered multiple analgesic medications (tramadol hydrochloride, pregabalin, celecoxib, and scopolamine butylbromide). These medications decreased the patient’s pain somewhat, and he reported that scopolamine butylbromide was most effective when the pain worsened. Because the patient’s symptoms were not relieved after trigger point treatment to the abdominal wall, we considered potential causes that might be associated with the location between the abdominal wall and visceral wall or related to other sources, including psychosocial, physiological, and other anatomical factors. We rechecked the abdominal CT scan for a suspected adhesion or abdominal hernia at the region of tenderness due to the prior surgical procedure, and we asked a radiologist to reevaluate the right upper abdomen in more detail. The radiologist confirmed a slight abnormality in the right upper abdomen and suggested the possibility of an adhesion around the surgical scar (Fig. 3). We referred the patient to a gastrointestinal surgeon for laparoscopic evaluation and adhesiolysis. The patient underwent additional investigations, including cholecystocholangiography and colonoscopy for suspected postcholecystectomy syndrome, biliary dyskinesia, or colon abnormality. However, no cause of the abdominal pain was identified. On laparoscopic evaluation, a broad adhesion was observed. Adhesiolysis was performed 6 months after the patient first visited our hospital. Figure 4a shows adhesion between the peritoneum and omentum, liver, and ascending colon; Fig. 4b shows the condition after adhesiolysis. One month after adhesiolysis, the patient’s right abdomen pain level during movement improved from 8 to 2–3 on a pain scale. Therefore, he was able to move with less pain, and he did not feel pain when rolling over. The result of Carnett’s test was negative. After the patient started walking around his house, he felt abdominal pain about 5 minutes after walking. Hence, he was afraid of recurrence of abdominal pain and felt a little depressed and frustrated because he was unable to return to work early. We recommended a gradual increase in activity. He went on a trip 4 months after the operation without problems due to abdominal pain and then resumed his job 6 months after the operation.

However, his abdominal pain deteriorated within 1 month after he resumed working. He presented with bleeding at the umbilicus, which was the laparoscopic port site, and abdominal incisional hernia was confirmed on the basis of CT. Repair of the abdominal incisional hernias and laparoscopic adhesiolysis were performed 8 months after the first operation. After the second operation, although it took time for some symptoms to improve because of surgical site infection, the patient’s symptoms were ultimately relieved, and he resumed his job again 5 months after undergoing the second operation. Although he reported mild abdominal pain and required analgesic medication, his weight decreased to 133 kg, and he was able to walk normally and work full-time, 2 years after he initially visited our hospital. The timeline of interventions and outcomes is shown in Additional file 1.

## Discussion

We report a patient with chronic abdominal pain induced by abdominal adhesion and the great challenge we experienced in identifying the origin of his pain. Specific symptoms and physical examinations were particularly valuable for determining the origin of the pain.

First, we suspected that the abdominal pain originated in the abdominal wall for the following three reasons:

1. Our patient presented with sharp, dull pain at the right abdomen and demonstrated a positive Carnett’s test. Although this test’s reliability has some limitations, it has a 78% sensitivity and 88% specificity for pain arising from an abdominal wall source [4]. Abdominal wall pain is characterized by being initially sharp, followed by a dull persistent ache, and is associated with a positive Carnett’s test [4].
2. With postural changes, such as standing up or turning over in bed, the patient’s pain was exacerbated. This situation also indicated abdominal wall pain. Srinivasan *et al.* [4] noted that pain may radiate diffusely when abdominal wall pressure increases or nerve traction occurs during standing, lifting, walking, or coughing.
3. The patient’s pain was chronic abdominal pain, which is a feature of abdominal wall pain that can easily become chronic [4].

Some studies suggest that in approximately 10–30% of all patients with chronic abdominal pain, the abdominal wall is the origin [4, 21]. More than 90% of patients with chronic abdominal pain have a positive Carnett’s test result, and the condition can be relieved by local anesthetic injection in the trigger points [22, 23]. Hence, we referred the patient to an anesthesiologist. When the anesthesiologist conducted a local injection with anesthetics, our patient’s abdominal pain was reduced but did not change substantially. Carnett [20] also indicated that there are three sensory layers of the abdominal wall that cause tenderness: the skin, muscles, and peritoneum. Finally, we speculated that the pain was associated with the abdominal wall and, in particular, considered that a peritoneal adhesion might also be involved because local anesthetic injections were less effective.

The patient’s pain was not only sharp but also dull, and it was relieved by scopolamine butylbromide rather than loxoprofen sodium hydrate. In addition, he experienced nausea. These features are associated with visceral pain. Scopolamine butylbromide is used to treat abdominal pain associated with cramps induced by gastrointestinal spasms as well as biliary acute spasm or renal colic [24, 25]. The pain emanated from around a postoperative scar, and a history of former abdominal surgery is the most important predictive factor for adhesion formation. In addition, the most common complications associated with adhesion are small bowel obstruction and chronic pain syndrome [26]. Chlamydial infection is known to bring about right upper quadrant abdominal pain due to intraabdominal adhesion (Fitz-Hugh–Curtis syndrome); however, chlamydial antibodies were negative in our patient. Many studies have addressed the association of chronic abdominal pain with intraperitoneal adhesion, particularly in the fields of obstetrics/gynecology and gastroenterological surgery [9, 27–29]. Kresch *et al.* [9] compared laparoscopic findings between 100 women with chronic pelvic pain who underwent the procedure to identify the source of pain and 50 asymptomatic women who underwent the procedure for tubal ligation. They found that 83% of women with chronic abdominal pain had abnormal pelvic organs, including adhesions, compared with 29% of asymptomatic women and concluded that while adhesions can cause pain, not all of them cause pain [9]. Adhesions were qualitatively different between the symptomatic and control groups, such that in patients with chronic pain and adhesions, there was restriction of motion or expansibility in one or more organs. Moreover, adhesions involving the parietal peritoneum or bowel are more likely to cause pain than adhesions involving other sites [9, 28]. Considering these studies, the history of abdominal surgery, severe abdominal pain during moving, positive Carnett’s test result, and ineffective local anesthesia made us suspect the cause of abdominal pain to be the peritoneum adhesion.

We referred the patient to the department of digestive surgery, where he underwent diagnostic laparoscopy and adhesiolysis 7 months after the onset of abdominal pain. That pain was relieved after the first laparoscopic adhesiolysis. Mueller *et al.* [28] recommended that patients with chronic abdominal pain lasting more than 6 months who have a history of laparotomy or pelvic inflammatory disease should undergo diagnostic laparoscopy. The application of laparoscopic adhesiolysis for chronic abdominal pain is controversial. Some studies have reported significant reduction in chronic abdominal pain after laparoscopic adhesiolysis [14, 30, 31]. Intermittent nausea and vomiting with colicky pain may be associated with intestinal adhesion, and in patients with a history of endometriosis or surgery, laparoscopic adhesiolysis is effective for the treatment of chronic pelvic pain in the long term [16]. In contrast, other studies have reported that pain reduction does not differ between laparoscopic adhesiolysis and diagnostic laparoscopy alone and therefore they did not recommend laparoscopic adhesiolysis as a treatment for adhesions in patients with chronic abdominal pain [31, 32]. In carefully selected patients with chronic abdominal pain, the positive effects of laparoscopic adhesiolysis can be sustained beyond 15 years after the surgery [33]. In one study, some patients (43%) underwent repeat adhesiolysis because of pain aggravation [14], and it took approximately 6 months for the pain to decrease from an average preoperative score of 8 to a postoperative score of 2 [15]. This patient improved for movements such as standing up and walking, as well as turning over in bed after laparoscopic adhesiolysis; therefore, he could resume his work. Abdominal adhesion does not always require laparoscopic adhesiolysis; however, if the adhesion causes restrictions in daily life activities, this procedure should be considered.

An association between obesity and ventral hernia has been reported [34], and our patient gained weight just before abdominal pain was noticed. Therefore, obesity may have been one factor in the etiology of the patient’s pain and his need for a second operation. We considered the possibility that weight gain was associated with this pathology. Increased obesity increases the amount of visceral fat, which may cause adhesions involving the parietal peritoneum or bowel to restrict motion or expansibility, ultimately causing more severe pain, as noted by Kresch *et al.* [9]. Therefore, we advised our patient to lose weight, and his symptoms did finally improve with the restoration of his original weight.

## Conclusions

Physicians should be aware of Carnett’s test as part of the evaluation of chronic undiagnosed abdominal pain, because many physicians are not familiar with this test and do not routinely perform it. It is difficult to detect the cause of chronic abdominal pain. If there is evidence of adhesion-induced abdominal pain based on the patient’s history, symptoms, and physical examination, especially when the patient has a positive result for Carnett’s test, physicians should carefully assess the findings of imaging modalities such as CT and consider referring the patient to a digestive surgeon for diagnostic laparoscopy and adhesiolysis.

## Additional file

Additional file 1: Timeline of interventions and outcomes. (DOCX 55 kb)

## Abbreviation

CT: Computed tomography
