# Supplementary material for: Adhesion-induced chronic abdominal pain: a case report on the diagnostic value of Carnett’s test
Source: J Med Case Rep. 2019 Apr 18;13:93. doi: 10.1186/s13256-019-2026-7 (PMC6474053; doi:10.1186/s13256-019-2026-7)
Supplement: Supplementary file 1 — Timeline of interventions and outcomes. (DOCX 55 kb) [file 13256_2019_2026_MOESM1_ESM.docx]

**Timeline of interventions and outcomes**

**Oct. 2015**

**Past Medical History**

**(Acute cholecystitis, cholecystectomy in 2011 32 years of age)**

Emergency surgery had done, but hospitalization was only two days.

After taking oral antibiotics for several days, he recovered and had no pain until 2015.

Gain weight: from 130 kg to 145 kg between January and February 2015. Right lateral abdominal pain starts in May. First hospital visit in Jun. No cause of abdominal pain was detected.

Positive for Carnett’s test, and a prior surgical scar of approximately 18 cm was apparent at the right subcostal region. Pain exacerbated to 8 on a pain scale when he moved, such as during standing up or rolling over simultaneously. Absent from work.

**Treatment (Intervention)**

Injection of 1% xylocaine 10 ml at a trigger point of the right lateral region led to about 30% relief in pain. Referral to anaesthesiologist and transversus abdominis plane block was performed, with negative result.

**Diagnostic Evaluations**

No inflammation, other laboratory findings were nonspecific, including liver/kidney function, blood glucose, urinary analysis and electrolytes. EKG is normal. Enhanced CT revealed bilateral renal stones and fatty liver.

Cholecystocholangiography and colonoscopy were negative.

**Jan. 2016**

**Jun. 2016**

**Sep. 2015**

**Jul. 2015**

First visit of our department

**Treatment (Intervention)**

Referral to gastrointestinal surgeon.

**Treatment (Intervention)**

laparoscopic evaluation detected broad adhesion, and adhesiolysis was performed.

Patient resumed working

Abdominal pain worsened and abdominal incisional hernia was confirmed on CT. Stopped working.

**Jul. 2016**

**Aug. 2016**

Bleeding at the umbilicus and abdominal pain worsened

**Sep. 2016**

**Treatment (Intervention)**

Repair of the abdominal incisional hernias and laparoscopic adhesiolysis

Surgical site infection

**Oct. – Dec. 2016**

**Jan. 2017**

Patient resumed working

**Ongoing Interventions and Follow-up**

Patient stopped going to the hospital regularly. He comes to the hospital for analgesics about once every three months.
